# Cancer Immunotherapy Update: FDA-Approved Checkpoint Inhibitors and Companion Diagnostics

**DOI:** 10.1208/s12248-021-00574-0

**Published:** 2021-03-07

**Authors:** Julianne D. Twomey, Baolin Zhang

**Affiliations:** Office of Biotechnology Products, Center for Drug Evaluation and Research, Food and Drug Administration, Silver Spring, Maryland 20993 USA

**Keywords:** cancer immunotherapy, immune checkpoint inhibitor, biomarker, companion diagnostic

## Abstract

Immune checkpoint inhibitors (ICIs) are considered a new standard-of-care across many cancer indications. This review provides an update on ICIs approved by the Food and Drug Administration (FDA), with focus on monoclonal antibodies that target the programmed cell death 1 (PD-1) or its ligand, PD-1 ligand 1 (PD-L1), including information on their clinical indications and associated companion diagnostics. The information is further discussed with strategies for identifying predictive biomarkers to guide the clinical use of PD-1/PD-L1-targeted therapies.

## INTRODUCTION

**Fig. 1:**
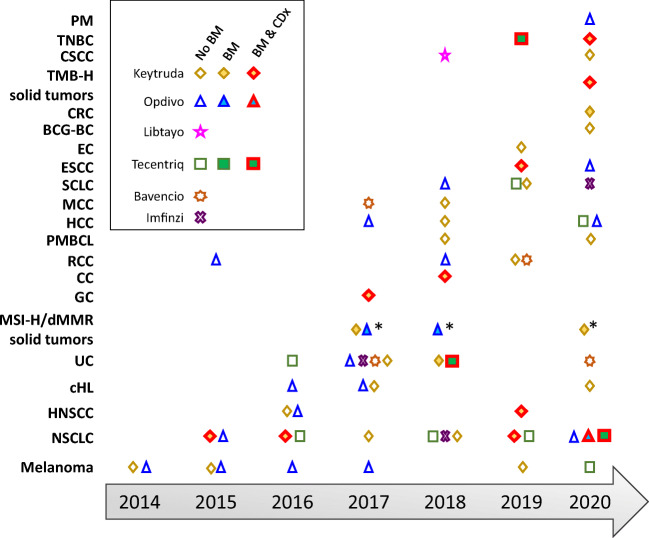
FDA approvals of PD-1/PD-L1 mAbs. As of December 2020, six anti-PD-1/PD-L1 mAbs have been approved with supplemental indications across 19 cancer types and two tissue-agnostic conditions. Shown are the approvals for each cancer indication, for Keytruda (pembrolizumab), Opdivo (nivolumab), Libtayo (cemiplimab), Tecentriq (atezolizumab), Bavencio (avelumab), and Imfinzi (durvalumab). Multiple approvals for a cancer indication within the same year are shown with only one symbol. The open symbols represent approvals without a biomarker (no BM). The full symbols represent approvals that incorporate a biomarker with an associated threshold for each indication (BM), which was measured using either a central laboratory test or complementary diagnostic that was not approved as a CDx for the drug. Symbols with a red outline represent approvals in which a companion diagnostic is indicated for biomarker measurement (BM + CDx). *: approval for MSI-H/dMMR colorectal cancer. PM, pleural mesothelioma; TNBC, triple-negative breast cancer; CSCC, cutaneous squamous cell carcinoma; TMB-H, tumor mutation burden high; CRC, colorectal cancer; BCG-BC, Bacillus Calmette-Guérin bladder cancer; EC, endometrial carcinoma; ESCC, esophageal squamous cell carcinoma; SCLC, small cell lung cancer; RCC, renal cell carcinoma; MCC, Merkel cell carcinoma; HCC, hepatocellular carcinoma; PMBCL, primary mediastinal large B cell lymphoma; CC, cervical cancer; GC, gastric cancer; MSI-H, microsatellite instability high; dMMR, mismatch repair-deficient; UC, urothelial carcinoma; cHL, classical Hodgkin’s lymphoma; HNSCC, head and neck squamous cell carcinoma; NSCLC, non-small cell lung cancer. Information on approvals and supplemental approvals was gathered from Drugs@FDA

The development of cancer immunotherapies, harnessing the immune system to restore anti-tumor immunity, has transformed the treatment of certain cancers. The first immune checkpoint inhibitor (ICI), an antibody targeting the cytotoxic T lymphocyte antigen 4 (CTLA4), was approved by the Food and Drug Administration in 2011 (1, 2). Since then, six more ICIs have been approved by the FDA, exclusively targeting the T cell co-inhibitory programmed cell death protein 1 (PD-1)/programmed cell death ligand 1 (PD-L1) signaling pathway (3, 4), with clinical indications across 19 different cancer types and two tissue-agnostic conditions (Fig. 1). While there is great promise in ICIs, only a small population of patients achieve a durable response to monotherapy. As a result, predictive biomarkers are used to “identify individuals who are more likely than similar individuals without the biomarker to experience a favorable or unfavorable effect from exposure to a medical product or an environmental agent.” (5) These markers, which are measured using validated *in vitro* assays, can aid in the enrichment of a patient population for clinical trials and for stratification of biomarker-positive and -negative patients. PD-L1 status on immune cells or tumor cells was considered to be one of the first potential predictive biomarkers for response to ICI treatment (6). Three of these approved ICIs targeting the PD-1/PD-L1 pathway (Keytruda (pembrolizumab), Opdivo (nivolumab), and Tecentriq (atezolizumab)) require the measurement of PD-L1. Identifying the appropriate biomarkers for these products requires understanding their mechanisms of action (MOAs) and tumor pathophysiology in individual patients with specific tumor types. This review will provide an update on the regulatory approvals of anti-PD-1/PD-L1 therapeutics along with their companion and complementary diagnostic devices.

## FDA-APPROVED ANTI-PD-1/PD-L1 THERAPIES

The standard of care for several cancer types currently includes treatment with monoclonal antibodies (mAbs) specific to PD-1 or PD-L1. PD-1 (CD279) is a co-inhibitory transmembrane protein that is expressed on antigen-stimulated T and B lymphocytes, natural killer (NK) cells, and myeloid suppressor dendritic cells (MDSCs). Following recognition of antigens or stimulation from cytokines, PD-1 is activated as a mechanism to modulate the intensity of the immune response (7). The engagement of PD-1 with its cognate ligands PD-L1 (B7-H1) or PD-L2 (B7-DC), which are widely expressed on tumor cells, results in the inhibition of T cell activation or proliferation and subsequently T cell exhaustion (3, 7, 8). While ICIs have demonstrated improved clinical efficacy, only a small proportion of patients respond to single-agent treatment. PD-L1 protein expression was the primary immuno-oncology biomarker, with the expression on immune cells and tumor cells being evaluated and quantified using immunohistochemistry (IHC) assays. The debate on whether PD-L1 expression levels are predictive of a response has been assessed through prospective or retrospective analysis, resulting in many ICI approvals with biomarker-independent treatment indications (1, 3). There remains a lack of universal predictive biomarker for patient selection for ICI treatment.

### Anti-PD-1 mAbs

Three anti-PD-1 antibodies have been approved by the FDA: pembrolizumab (Keytruda), nivolumab (Opdivo), and cemiplimab (Libtayo).

#### Pembrolizumab (Keytruda)

Pembrolizumab, a humanized IgG4 antibody against PD-1, was initially approved by the FDA in September 2014 following results from the KEYNOTE-001 clinical trial (NCT01295827), studying patients with unresectable or metastatic melanoma and patients with non-small cell lung cancer (NSCLC). These cancer types were chosen as there were previously seen high levels of PD-L1 expression (9, 10). The approval was specified for the treatment of patients with unresectable or metastatic melanoma and disease progression after receiving ipilimumab and, in patients with BRAF^V600^ mutation, a BRAF inhibitor (11). Improvements were seen in overall response rate (ORR) and duration of response (12). This was later expanded to include treatment of patients with melanoma with involvement of lymph nodes following complete resection.

The incorporation of threshold inclusion criteria based on the expression level of PD-L1 protein was approved in 2015, for the treatment of patients with PD-L1-positive NSCLC as determined by an FDA-approved test along with the approval of the PD-L1 IHC 22C3 pharmDx (Dako). In the NSCLC cohort of the trial, patients were analyzed for their PD-L1 tumor proportion score (TPS), which is the percentage of tumor cells that express PD-L1 identified using IHC analysis (13, 14). Patients were separated into cohorts based on expression levels of < 1% TPS, 1–49% TPS, and ≥ 50% TPS, and considered positive if they had a TPS ≥ 1% (15). Patients with a TPS ≥ 1% had an increased ORR compared to those < 1%, with the highest benefit in the patients with ≥ 50% TPS (13, 14). The indication for metastatic NSCLC was expanded in 2016, to include patients with TPS ≥ 1% with disease progression on or after platinum-containing chemotherapy and metastatic NSCLC with high PD-L1 expression (TPS ≥ 50%) with no EGFR or ALK genomic tumor aberrations, and no prior systemic chemotherapy treatment. An improved overall survival rate was seen in patients with high PD-L1 expression (16, 17). Not all lung cancer indications require a PD-L1 protein measurement, including the first-line treatment of patients with squamous or non-squamous NSCLC as a single agent or in combination with carboplatin and either paclitaxel or nab-paclitaxel (18, 19) or patients with SCLC with disease progression on or after platinum-based chemotherapy, and at least one other prior line of therapy (20, 21). In these clinical studies, patients demonstrated benefit regardless of the level of PD-L1 expression.

In 2016, pembrolizumab was approved for the treatment of patients with recurrent or metastatic squamous cell carcinoma of the head and neck (HNSCC). In 2019, an additional indication for HNSCC was approved, for patients whose tumors express PD-L1 for a combined positive score of more than 1 (CPS ≥ 1) as determined by an FDA-approved test (22). The CPS determines the amount of PD-L1-positive cells that are within the tumor, including the tumor cells, lymphocytes, and macrophages relative to the total viable cell counts. Patients with a positive PD-L1 expression (CPS > 1) derived benefit, and those patients who expressed a CPS > 20 were found to have the most benefit, with an increase in OS when treated with pembrolizumab with chemotherapy compared to cetuximab with chemotherapy (22). Approvals for indications for locally advanced or metastatic gastric or gastroesophageal junction carcinoma (23–25) and recurrent or metastatic cervical cancer (26, 27) both require the determination of a PD-L1 score of CPS ≥ 1 for treatment, while the approvals for locally advanced or metastatic urothelial carcinoma (28), locally advanced or metastatic squamous cell carcinoma of the esophagus (29), and locally recurrent unresectable or metastatic triple-negative breast cancer (TNBC) require the determination of a PD-L1 score of CPS ≥ 10 for treatment (30–32).

In 2017, a novel indication was approved, which included any solid tumor which had microsatellite instability (MSI-H) or dMMR status. This approval was the first time a cancer treatment was approved based on a common biomarker across cancer types, regardless of the cancer of origin (33). The data was collected from single-arm cohorts of clinical trials, for a pooled analysis, in which patient samples were analyzed retrospectively using a central laboratory-developed PCR test, and patients with an MSI-H had a significantly higher ORR and increased duration of response compared to patients with microsatellite-stable (MSS) tumors (25, 33). This was expanded to include patients with unresectable or metastatic, MSI-H or dMMR solid tumors or metastatic MSI-H, or dMMR colorectal cancer that has progressed following treatment with a fluoropyrimidine, oxaliplatin, and irinotecan (34). Mutations in the mismatch repair genes (MLH1, MSH2, MSH6, and PMS2) can lead to MSI due to errors in the DNA microsatellites. Tumors with high levels of mismatch repair mutations are commonly associated with higher levels of neoantigen production (33), rendering the tumors susceptible to the ICI therapy.

In 2020, a new indication was added with a companion diagnostic, for the treatment of patients with tumor mutational burden high (TMB-H) cancer, as determined by an FDA-approved test (35, 36). TMB is defined by the number of somatic mutations per megabase (Mb) across an interrogated genomic sequence (35). Within the Keynote-158 (NCT02628067) clinical trial, retrospective analysis was performed on tumor samples and the TMB of ≥ 10 or ≥ 13 mutations (mut) per Mb was analyzed by the Foundation One CDx (36). Patients with TMB-H (≥ 10 mut/Mb) were found to have an ORR of 29% and patients with TMB ≥ 13 mut/Mb achieved an ORR of 37%. The higher mutational burden within a tumor is expected to correspond to a higher level of immunogenic neopeptides that would drive T cell-mediated anti-tumor immunity (35–37).

Several additional indications without biomarker requirements were approved over the past 5 years, including indications for the treatment of adult and pediatric patients with refractory classical Hodgkin’s lymphoma (38, 39), locally advanced or metastatic urothelial carcinoma for patients who are not eligible for cisplatin-containing chemotherapy or who have had disease progression during or following platinum-containing chemotherapy (40, 41), mediastinal large B cell lymphoma (42, 43), hepatocellular carcinoma (44), Merkel cell carcinoma (45, 46), patients with advanced renal cell carcinoma, recurrent or metastatic cutaneous squamous cell carcinoma (22, 47), and patients with Bacillus Calmette-Guerin unresponsive, high-risk, non-muscle invasive bladder cancer (48).

#### Nivolumab (Opdivo)

Nivolumab, an IgG4 mAb against PD-1, was approved following the pivotal trial CheckMate-037, on December 22, 2014, for the treatment of patients with unresectable of metastatic melanoma who have experienced disease progression following ipilimumab and, if BRAF^V600^ mutation-positive, a BRAF inhibitor. Between 2015 and 2020, new indications were approved for the treatment of patients with metastatic NSCLC with progression on or after platinum-based chemotherapy (49, 50), for treatment in combination with ipilimumab or as a single agent for unresectable or metastatic melanoma patients (51), for the treatment of patients with advanced renal cell carcinoma (52), classical Hodgkin’s lymphoma (53), recurrent or metastatic squamous cell carcinoma of the head and neck (HNSCC) (54), locally advanced or metastatic urothelial carcinoma, hepatocellular carcinoma (55), metastatic SCLC (56), metastatic or recurrent NSCLC (57), esophageal squamous cell carcinoma (ESCC) (58), and for patients with unresectable malignant pleural mesothelioma (59, 60).

In the CheckMate 017 phase 3 clinical trial studying squamous cell NSCLC, PD-L1 expression ( ≥ 1%, ≥ 5%, ≥ 10%) was used for retrospective analysis and stratification to determine efficacy, though expression levels were not found to be prognostic or predictive of benefit (49, 61). In the CheckMate 057 and CheckMate 063, similar retrospective stratification was performed, which demonstrated that PD-L1 expression was predictive of benefit to treatment with nivolumab (61, 62). PD-L1 positivity was determined as ≥ 5%, as previous studies did not distinguish a greater response when a threshold of 1% was used (50). Patients who had PD-L1-positive tumors had more objective response compared to patients with PD-L1-negative tumors, though this was not considered to be significant due to sample size.

The CheckMate 275 clinical trial, studying nivolumab as a first-line treatment of patients with metastatic or surgically unresectable urothelial carcinoma, determined PD-L1 expression at screening of patients using the Dako PD-L1 IHC 28-8 pharmDx kit, though this was not used as inclusion criteria (63). Patients experienced benefit from the treatment, irrespective of PD-L1 expression (63). Patient samples were further evaluated in the 2-year follow-up for novel biomarker discovery, in which retrospective analysis demonstrated that patients with higher TMB had improved ORR and OS, which was further improved when TMB was combined with PD-L1 status (64). TMB stratification was divided into three groups, with low < 85, medium 85–169, and high ≥ 170 missense somatic mutations per tumor (64). The TMB levels and PD-L1 expression were not correlated.

PD-L1 status was used to stratify the patients with resected stage IIIB–C or stage IV melanoma in the CheckMate 238 (51). Patients were randomized to receive either ipilimumab or nivolumab and stratified based on disease stage and PD-L1 status (≥ 5% of tumor cells compared to < 5 % or indeterminate staining). Recurrence-free survival was higher in patients with a higher PD-L1 expression, though all patients experienced greater benefit when treated with nivolumab compared to ipilimumab (51). In May 2020, treatment with nivolumab in combination with ipilimumab was approved for first-line treatment of patients with metastatic or recurrent NSCLC with no EGFR or ALK genomic tumor aberrations (65). Patients were enrolled in the CheckMate 9LA trial regardless of PD-L1 status, and randomized to receive nivolumab with ipilimumab and chemotherapy or chemotherapy alone, with cohorts stratified based on PD-L1 status (< 1% *vs* ≥ 1%) (57). Clinical benefit was seen across all groups that were treated with the ICI combination, regardless of biomarker status. Stratification by biomarker was also performed in the Attraction-3 clinical trial, in which patients with unresectable advanced, recurrent, or metastatic ESCC were enrolled regardless of PD-L1 status. PD-L1 expression was determined by the PD-L1 IHC 28-8 pharmDx assay at a central testing laboratory, and patients were randomized using PD-L1 ≥ 1% or < 1% or indeterminate staining. No clinical benefit was seen that was dependent on PD-L1 status, with all patients treated with nivolumab having significant improvement in OS (58). In May 2020, the inclusion of a biomarker was approved for nivolumab in the treatment of adult patients with metastatic or recurrent NSCLC whose tumors express PD-L1 (≥ 1%) as determined by an FDA-approved test (66). PD-L1 status was measured using the PD-L1 IHC 28-8 pharmDx assay and patients were randomized 1:1:1 to receive either nivolumab plus ipilimumab, nivolumab monotherapy, or chemotherapy. Patients with PD-L1-positive tumors treated with the combination treatment reported a longer overall survival rate and a longer median duration of response (66). The addition of the biomarker inclusion criteria was accompanied by the approval of the PD-L1 IHC 28-8 pharmDx assay as a companion diagnostic for the indication of Opdivo (67). Patient samples were also screened for tumor mutational burden for exploratory biomarker analysis, though no correlation was seen for TMB-high *vs*. TMB-low with overall survival benefit. TMB and PD-L1 status also did not report a correlation with benefit in this patient population (66).

Indications for nivolumab treatment for patients with MSI-H or dMMR metastatic colorectal cancer as a single agent were approved in 2017 and in combination with ipilimumab in 2018 (68). MSI/dMMR status was determined either by PCR or IHC using a central testing laboratory assay. PD-L1 status was also determined using the Dako 28-8 pharmDx assay (≥ 1% or < 1%) (68, 69). Improved rate of disease control and ORR was reported for patients with dMMR/MSI-H when treated with nivolumab, regardless of PD-L1 status (68).

#### Cemiplimab-rwlc (Libtayo)

Cemiplimab is a human IgG4 anti-PD-1 mAb that was approved in 2018 for the treatment of patients with metastatic cutaneous squamous cell carcinoma (CSCC) or locally advanced CSCC who are not candidates for curative surgery or radiation (70). This cancer type was studied due to known high mutational burden. A response was seen in half of the patients in the pivotal phase II study with an acceptable safety profile (70, 71).

### Anti-PD-L1 mAbs

Three anti-PD-L1 antibodies have been approved by the FDA: atezolizumab (Tecentriq), durvalumab (Imfinzi), and avelumab (Bavencio).  

#### Atezolizumab (Tecentriq)

Atezolizumab, a humanized anti-PD-L1 mAb, was approved in 2016 for the treatment of patients with advanced or metastatic urothelial carcinoma. PD-L1 expression was evaluated on tumor specimens prospectively using the Ventana PD-L1 (SP142) Assay, using the threshold cutoff of more than 5% of the tumor area having PD-L1-positive tumor-infiltrating immune cells (IC) (72). This threshold only includes the PD-L1 positivity of the immune cells within the tumor microenvironment. PD-L1 expression was defined based on expression status of the immune cells and separated into cohorts of IC0 (< 1%), IC1 (≥ 1% but < 5%), and IC2/3 (≥ 5%). Response rate was seen to correlate with the increased expression of PD-L1 status on ICs (72). Genomic profiling was also conducted for exploratory biomarker analysis using the FoundationOne panel (72). Treatment with atezolizumab resulted in improved survival, with higher levels of PD-L1 expression on immune cells, though not with tumor cells, or higher TMB associated with higher response rate (73, 74). PD-L1 expression was then incorporated into FDA labeling in 2018 following the IMvigor210 clinical trial, to select patients who should receive Tecentriq treatment (74, 75). Tumor specimens were prospectively evaluated using the Ventana PD-L1 (SP142) assay and patients with high levels of PD-L1 expression had improved PR, CR, and ORR.

Clinical trials studying atezolizumab treatment in advanced cancers (NSCLC, melanoma, renal cell carcinoma, colorectal, gastric, and HSCC) led to approved indications due to increased response rates in patients treated with atezolizumab (76). Biomarker inclusion was studied in most of these trials but was not initially included in the FDA labeling. In NSCLC patients, there was a correlation between PD-L1 expression and response to treatment, in which patients were stratified based on PD-L1 status on tumor-infiltrating immune cells and tumor cells and randomized to receive either atezolizumab or docetaxel (76). Patients were classified as having high PD-L1 expression if more than 50% of their tumor cells or 10% of their immune cells expressed PD-L1 membranous staining. PD-L1 positivity correlated with improved OS, PFS, and ORR when treated with atezolizumab as a single agent (77–79). In May 2020, following the IMpower110 (NCT02409342) clinical trial, the inclusion criteria of high PD-L1 expression ≥ 50% of tumor cells or ≥ 10% of tumor-infiltrating immune cells as defined by an FDA-approved device were approved for the treatment of adult metastatic NSCLC with no EGFR or ALK genomic aberrations (78). The overall survival rate was 20.2 months for patients with PD-L1 high-expressing tumors treated with atezolizumab compared to 13.1 months for patients treated with chemotherapy, and patients with PD-L1-positive tumors (tumor proportion score > 1%) had an OS of 17.8 months in the atezolizumab-treated cohort compared to 14.1 months in the chemotherapy-treated (78). In March 2019, the FDA approved the new indication for treatment with atezolizumab in combination with nab-paclitaxel for the treatment of adult patients with unresectable locally advanced or metastatic TNBC whose tumors express PD-L1 (IC ≥ 1% of tumor area) as determined by an FDA-approved test. This was the first ICI approval for the treatment of patients with breast cancer, with significantly longer PFS compared to the placebo arm (80).

While PD-L1 status was shown to correlate with improved response rates in some clinical studies, the evaluation of PD-L1 expression is not always performed, depending on the study population and the primary endpoints evaluated. In patients with ES-SCLC, the IMpower133 clinical trial, studying the treatment of atezolizumab in combination with carboplatin and etoposide, the primary endpoints of PFS and OS were met, with improved survival for patients treated with atezolizumab without evaluation of PD-L1 status (81). The IMspire150 clinical trial stratified patients with BRAF^V600^ mutation-positive advanced or metastatic melanoma using lactate dehydrogenase concentration, demonstrating significantly increased PFS in patients treated with atezolizumab (82). The IMbrave150 clinical trial reported significant improvements in the OS and PFS in the atezolizumab-treated patients with hepatocellular carcinoma as a first-line treatment (83).

#### Durvalumab (Imfinzi)

Durvalumab is an IgG1κ anti-PD-L1 mAb that was first approved in 2017 for the treatment of locally advanced or metastatic urothelial carcinoma. PD-L1 expression was prospectively determined in patients with solid tumors using the Ventana PD-L1 (SP263) assay, in which expression levels were classified as PD-L1 high (if ICs involve > 1% of the tumor area, TC ≥ 25% or IC ≥ 25%; if ICs involve < 1% of the tumor area, TC ≥ 25% or IC = 100%) or PD-L1 low/negative (84, 85). In the urothelial carcinoma cohort, the PD-L1 high patients experienced an improved disease control rate but patients treated with durvalumab experienced response regardless of PD-L1 status. In 2018, an additional indication was approved for durvalumab, for the treatment of adult patients with ES-SCLC in combination with etoposide and either carboplatin or cisplatin, which reported significant improvement in OS compared to the control group (86).

#### Avelumab (Bavencio)

Avelumab is a fully human IgG1 anti-PD-L1 mAb that was approved under accelerated approval in 2017 for the treatment of patients with metastatic Merkel cell carcinoma (MCC), in which patients’ response to the therapy was not dependent on PD-L1 positivity. This was the first treatment for mMCC, with an ORR of 46.7% (87). Avelumab was then approved for the treatment of urothelial carcinoma patients and for the treatment of patients with advanced renal cell carcinoma (88). Though PD-L1 expression was evaluated and demonstrated an increase in ORR correlated with expression levels, the ORR was achieved in all expression cohorts. OS was not found to be correlated with PD-L1 expression; therefore, the protein expression was not considered predictive (88).

## FDA CLEARED DIAGNOSTICS FOR USE WITH ANTI-PD-1/PD-L1 THERAPEUTICS

**Fig. 2:**
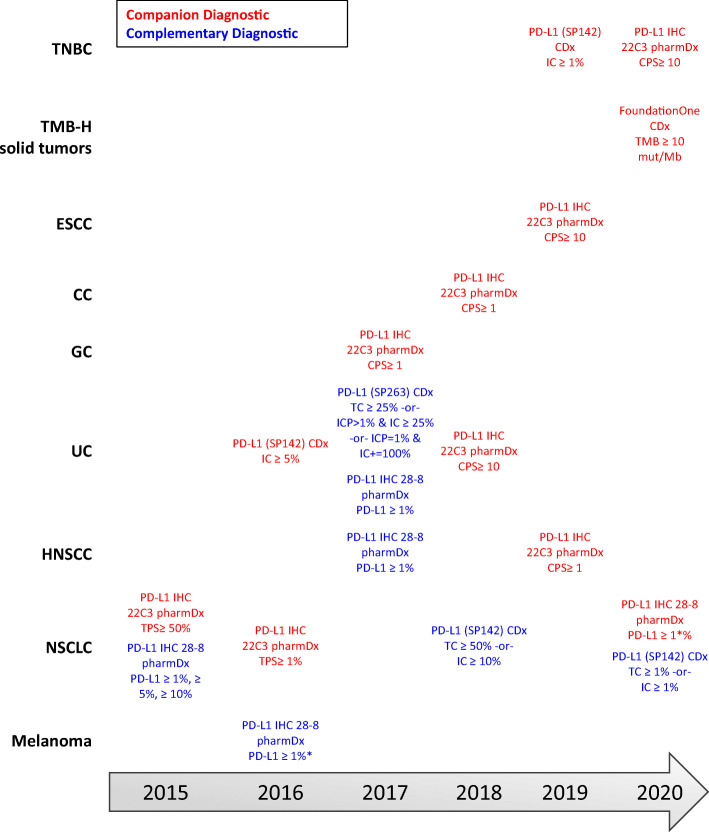
FDA approvals of companion and complementary diagnostic assays. As of December 2020, there are five companion diagnostics that have been approved to identify patients across seven tissue types and one tissue-agnostic condition who may benefit from treatment with an anti-PD-1/PD-L1 mAb. Shown are the approvals for each companion (indicated in red text) and complementary (indicated in blue text) device with the associated threshold per cancer type. Tumor proportion score (TPS) measured the membranous staining of tumors cells and is reported as a percentage of the total viable tumor cells. Combined proportion score (CPS) measures the membranous staining of tumor cells, lymphocytes, and macrophages and is reported as a percentage of the total viable tumor cells and multiplied by 100. PD-L1 percentage (as measured by the 28-8 pharmDx) reports the number of tumor cells with complete circumferential or partial linear plasma membrane staining of PDL1 out of 100 viable tumor cells. IC and TC measure the proportion of tumor area occupied by PD-L1 expressing tumor-infiltrating immune cells (IC) or the percentage of PD-L1-positive tumor cells (TC) and is reported as a percentage of the tumor area. The percentage of immune cells present (ICP) is reported as the percentage of tumor area occupied by any tumor-associated immune cells. *The melanoma indication was withdrawn from the PD-L1 IHC 28-8 pharmDx label on 07 March 2019. TNBC, triple-negative breast cancer; TMB-H, tumor mutation burden high; ESCC, esophageal squamous cell carcinoma; CC, cervical cancer; GC, gastric cancer; UC, urothelial carcinoma; HNSCC, head and neck squamous cell carcinoma; NSCLC, non-small cell lung cancer. Information was collected from the “List of Cleared or Approved Companion Diagnostic Devices (In Vitro and Imaging Tools) (https://www.fda.gov/medical-devices/vitro-diagnostics/list-cleared-or-approved-companion-diagnostic-devices-vitro-and-imaging-tools)

The use of a companion or complementary diagnostic device for PD-L1 expression levels has been included in many clinical trials and FDA labeling across cancer types. While a companion diagnostic device is required for the therapeutic product’s safe and effective use, a complementary test is performed to provide information that is clinically meaningful and will aid in the decision regarding treatment (89). The first companion diagnostic for an ICI targeting the PD-1/PD-L1 signaling pathway was approved in 2015 through the Premarket Approval process, for use in identifying NSCLC patients for treatment with pembrolizumab (Fig. 2) (90). Since the initial approval, the PD-L1 IHC 22C3 pharmDx assay approval has been extended to patients with gastroesophageal and gastroesophageal junction cancer, cervical cancer, UC, HNSCC, and TNBC. As previously described, the device uses the TPS to identify NSCLC patients who are PD-L1-positive (TPS ≥ 1%) and CPS for the additional biomarker-dependent indications, either CPS ≥ 1 or CPS ≥ 10. TPS identifies the percentage of PD-L1-positive tumor cells relative to the viable tumor cells within the sample, whereas CPS identifies the PD-L1-positive cells, including tumor cells, lymphocytes, and macrophages (91, 92).

The Ventana PD-L1 (SP142) assay was specifically developed for use with atezolizumab and was tested in the pivotal studies that led to the therapeutics approval and the approval of the assay as a complementary diagnostic (93). This device uses IHC to determine partial or circumferential membrane or associated cytoplasmic staining of IC or TC. The approval was updated to include the assay as a companion diagnostic for the identification of patients with urothelial carcinoma (≥ 5% IC cutoff) (94), TNBC (≥ 1% IC cutoff) (95), and NSCLC (≥ 50% TC cutoff or ≥ 10% IC) with the approved therapeutic product labeling. The Ventana PD-L1 (SP263) assay was developed for clinical trial enrollment of patients intended for treatment with durvalumab, to determine the percentage of tumor cells and tumor-associated immune cells with any membrane staining of PD-L1 and is used as a complementary diagnostic.

The approval of the PD-L1 IHC 28-8 pharmDx as a companion diagnostic intended for use in the detection of PD-L1 protein to identify NSCLC patients for treatment with nivolumab in combination with ipilimumab was granted in May 2020 (67). The assay was originally developed for use with clinical trials with nivolumab and was approved as a complementary diagnostic device for second-line treatment of NSCLC, as patients treated with nivolumab demonstrated increased response rate regardless of PD-L1 status (96). PD-L1 expression as a predictive biomarker was evaluated using retrospective analysis, in which there was a statistically significant difference in OS of PD-L1-positive patients treated with nivolumab in combination with ipilimumab compared to docetaxel treatment. The device may also be used to determine the PD-L1 protein expression in patients with SCCHN and UC, but as a complementary diagnostic device.

Comparability across assays and their diagnostic use has been discussed, as approvals indicate specific devices for the different therapeutics with varying biomarker thresholds (96). The consistency in identifying PD-L1-positive patients and the concordance across devices has been studied in the Blueprint PD-L1 IHC Assay Comparison Project, in which concordance was seen across the 28-8, 22C3, and SP263 devices, though not for the SP142 assay (97, 98). As PD-L1 positivity is still being evaluated as a predictive biomarker in clinical trials, in which patients with negative or non-evaluable tumor samples have also demonstrated a response, additional biomarkers are being assessed to determine their correlation with response rates and to better identify those patients who will respond.

In the Keynote-158 clinical trial, patients with solid tumors were enrolled and a tumor sample was taken for biomarker analysis. These samples were assessed by the FoundationOne CDx assay to determine the tissue tumor mutation burden (tTMB), with a threshold cutoff of more than 10 mutations per megabase as determined by whole exome sequencing (36, 99). The association between the efficacy of pembrolizumab, as determined by CR or PR and high tTMB resulted in the approval of the assay as a companion diagnostic to determine treatment with pembrolizumab in patients with TMB-high solid tumors. The approval of the FoundationOne CDx as a companion diagnostic also indicates there is a universal threshold for TMB across tumor types in determining treatment with pembrolizumab (99).

## EXPANDED BIOMARKER DISCOVERY AND POTENTIAL COMBINATION THERAPIES

To better identify the population who will most benefit from ICI treatment and those who may be susceptible to immune-related adverse events, strategies are being implemented to expand the use of ICIs and develop novel biomarkers using proteomic, genomic, and transcriptomic analysis. This involves a deeper understanding of evolving resistance mechanisms, primary resistance, and the factors that impact ICI efficacy. As described above, the use of PD-L1 as a predictive biomarker to identify those patients who are most likely to benefit from ICI treatment remains difficult due to different assays used for each therapeutic, difference in threshold cutoffs across indications, tumor heterogeneity within and across patient populations, the diversity of patients’ treatment history, and the dynamic status of the tumor microenvironment. In some cases, a single-parameter biomarker (e.g., PD-L1) may not be sufficient to accurately stratify patients for ICI therapy (100).

Strategies being explored for the development of novel biomarkers also include further understanding of the tumor microenvironment. The cancer immunity cycle is initiated when the accumulation of genetic mutations within cancer cell results in the production of neoantigens, which are able to bind to major histocompatibility complex (MHC) molecules on the cancer cell plasma membrane (101). As cancer cells die during tumor growth, neoantigens are released and captured by dendritic cells (DCs) or antigen-presenting cells (APCs), which migrate to the lymphoid organs. The DCs present the antigens to T cells to prime and activate the T cells using co-stimulatory (CD28, CD80, CD86) and co-inhibitory molecules (PD-L1, CTLA4), to regulate the tumor-specific T cells and encourage the T cells to become effector cells (101). The T cells then target the foreign antigen/tumor cells through binding of T cell receptor (TCR) to the antigen-bound MHCs on the cancer cells, leading to cell lysis and further antigen release (101). Identification of these neoantigens or how these proteins are involved in the cancer immunity cycle may help identify novel predictive biomarkers.

Exploring how biomarkers interact may also aid in the design of combination strategies, to maximize their benefit (99). Various clinical trials are studying the sequential treatment of ICIs either prior to or following chemotherapies, to determine if this treatment can turn “cold” non-immunogenic tumor to a “hot” tumor, which would respond to ICI treatment (NCT00527735, NCT02499367). The goal of these combinations is to modulate the immune suppressive microenvironment and initiate tumor cell death, recruiting effector T cells to the tumor and increasing the efficacy of the ICIs. Using retrospective analysis, potential biomarkers that may correlate with increased response rate to ICIs include the neutrophil to lymphocyte ratio (NLR) (102) or an absolute eosinophil count (103). Due to the dynamic nature of PD-1/PD-L1, it has been challenging to detect the changing PD-1/PD-L1 expression using solid tumor tissue biopsy during tumor progress or treatment. As a result, several trials are also incorporating liquid biopsies to monitor the soluble PD-1/PD-L1 in the peripheral blood (104, 105).

Combination therapies are in the limelight for PD-1/PD-L1 clinical trials (106). Ongoing clinical trials are testing combination and sequential therapies, such as additional immuno-oncology treatments that target parallel signaling pathways, chemotherapies known to increase antigen release, or radiotherapies (106, 107). PD-L1 expression is known to change following chemotherapy, radiation therapy, and several targeted therapies (108, 109). Understanding how these therapies may work together may require a continuous monitoring of biomarkers to aid in treatment decisions, to evaluate whether a first-line treatment switches a tumor from TMB-low to TMB-high or modulates PD-L1 expression above a threshold cutoff. Determining the optimal sequence, dosing, and timing of combination therapies to modify the tumor microenvironment while evading acquired resistance may further expand the use of ICIs. Examination of the changing tumor microenvironment during these combinations, with understanding the contribution of genomic or proteomic biomarkers to response rates, will hopefully improve patient response and expand the potential patient population who will benefit from these therapies.

## CONCLUSION

With the rapid growth of PD-1/PD-L1 blockade in clinical use, an effective biomarker to identify patients who are likely or unlikely to benefit from these therapies becomes increasingly necessary. The currently available biomarkers (PD-L1 expression, TMB-H, dMMR/MSI-H) are helpful to the selection of patients for PD-1/PD-L1 therapy in several tumor types. However, due to the intra- and inter-tumoral heterogeneity, there are still many challenges for their expanded use across different products and tumor types. PD-L1 thresholds that determine a biomarker-positive patient are inconsistent across different assays, differing in thresholds both within and across tumor types. Biomarker positivity is dependent on the assay, which varies in measuring PD-L1 expression on tumor cells alone or in conjunction with tumor-infiltrating immune cells and is specific to each immuno-oncology product. Harmonization of the different diagnostic assays and their scoring metrics is critical to provide patients with consistent information regarding the selection of optimal treatment strategies. The tissue-agnostic signature such as TMB-H and dMMR/MSI-H holds promise to guide the prescription of PD-1/PD-L1 therapy, but their predictive value is limited by the lack of pre-defined criteria for each product in a specific tumor. Given the complexity of the immune system, a single-parameter biomarker (e.g., PD-L1) may not be sufficient to accurately predict therapeutic benefit in individual patients. Composite biomarkers of multiple variables may be able to better predict patient outcomes. Regardless, prospective randomized trials are required to establish the roles of predictive biomarkers in specific clinical settings.

## Declarations

### Disclaimer

This article reflects the views of the authors and should not be construed to represent FDA’s views or policies.
